# Golgi Complex Dynamics and Its Implication in Prevalent Neurological Disorders

**DOI:** 10.3389/fcell.2019.00075

**Published:** 2019-05-07

**Authors:** Mario O. Caracci, Luz M. Fuentealba, María-Paz Marzolo

**Affiliations:** Departamento de Biología Celular y Molecular, Facultad de Ciencias Biológicas, Pontificia Universidad Católica de Chile, Santiago, Chile

**Keywords:** Golgins, GOPs, CLASP2, neurodegeneration, epilepsy, LRRK2, Reelin, synaptic activity

## Abstract

Coupling of protein synthesis with protein delivery to distinct subcellular domains is essential for maintaining cellular homeostasis, and defects thereof have consistently been shown to be associated with several diseases. This function is particularly challenging for neurons given their polarized nature and differential protein requirements in synaptic boutons, dendrites, axons, and soma. Long-range trafficking is greatly enhanced in neurons by discrete mini-organelles resembling the Golgi complex (GC) referred to as Golgi outposts (GOPs) which play an essential role in the development of dendritic arborization. In this context, the morphology of the GC is highly plastic, and the polarized distribution of this organelle is necessary for neuronal migration and polarized growth. Furthermore, synaptic components are readily trafficked and modified at GOP suggesting a function for this organelle in synaptic plasticity. However, little is known about GOPs properties and biogenesis and the role of GOP dysregulation in pathology. In this review, we discuss current literature supporting a role for GC dynamics in prevalent neurological disorders such as Alzheimer’s disease, Parkinson’s disease, Huntington’s disease, and epilepsy, and examine the association of these disorders with the wide-ranging effects of GC function on common cellular pathways regulating neuronal excitability, polarity, migration, and organellar stress. First, we discuss the role of Golgins and Golgi-associated proteins in the regulation of GC morphology and dynamics. Then, we consider abnormal GC arrangements observed in neurological disorders and associations with common neuronal defects therein. Finally, we consider the cell signaling pathways involved in the modulation of GC dynamics and argue for a master regulatory role for Reelin signaling, a well-known regulator of neuronal polarity and migration. Determining the cellular pathways involved in shaping the Golgi network will have a direct and profound impact on our current understanding of neurodevelopment and neuropathology and aid the development of novel therapeutic strategies for improved patient care and prognosis.

## Introduction

The Golgi complex (GC) is composed of distinct compartments or cisternae: the *cis-*, *medial-* and *trans-*Golgi and the *trans*-Golgi network (TGN) (Chen et al., 2017). The *cis*-Golgi, located immediately after the endoplasmic reticulum (ER), is where proteins are sorted based on carboxyl terminal signal peptides to either continue moving through the Golgi cisternae or return to the ER, or, in the case of lysosomal enzymes, be tagged with mannose-6-phosphate to be directed to the lysosomes. The *medial*-Golgi is enriched in glycosyl-transferase enzymes and conducts several post-translational modifications of recently translated proteins. The *trans*-most Golgi cisterna branches toward a tubular and reticulating compartment called the TGN, considered the central cargo sorting station of the cell (Guo et al., 2014). Both clathrin- and non-clathrin coated vesicles, as well as tubules, are formed at the TGN to deliver lipid and proteins to their final destination, mainly plasma membrane and endosomal compartments (Lowe, 2011; Guo et al., 2014). Golgi cisternae are laterally linked to form the Golgi ribbon structure. The stability of this complex structure and the maintenance of cisternae-specific protein makeup largely depends on resident structural proteins and their interaction with both microtubules and the actin cytoskeleton (Lowe, 2011).

Excellent reviews have addressed the biology of the GC in an array of cell types (Hanus and Ehlers, 2008; Nakano and Luini, 2010; Lowe, 2011; Ramírez and Couve, 2011; Muschalik and Munro, 2018). However, little attention has been given to the role of GC in pathological conditions associated with the central nervous system (CNS). Increased GC fragmentation has been reported in several neurological disorders, including Alzheimer’s disease (AD) (Joshi et al., 2015), amyotrophic lateral sclerosis (ALS) (Sundaramoorthy et al., 2015), Creutzfeldt-Jakob disease (Sakurai et al., 2000), and Parkinson’s disease (PD) (Gosavi et al., 2002), among others. Compellingly, this fragmentation is often observed at early stages of the disease thus making it unlikely that GC fragmentation is a result of apoptotic cell death (Gosavi et al., 2002; Liazoghli et al., 2005; van Dis et al., 2014).

In the present review, we summarize relevant information regarding the regulation of GC dynamics in neurons and associate it with common cellular and molecular defects identified in neurodevelopmental and neurodegenerative disorders. First, we describe the distinct role and organization of the GC in neurons, where it participates of relevant developmental processes such as neuronal polarity and migration. Afterward, we discuss the role of Golgins in CNS development and disease. We then address the convergence of well-known neurological disorders with abnormal GC architecture observed therein. Finally, we link all cellular pathways discussed in the previous sections with known cellular outcomes of Reelin signaling activation, a cell signaling pathway consistently associated with CNS development that includes neuronal polarity and Golgi/cytoskeletal dynamics (Santana and Marzolo, 2017).

## Architecture and Function of the GC in Neurons

The role of the GC in polarized cells such as neurons is particularly challenging given the differential protein composition of each neuronal compartment and the long distances that newly synthesized and modified proteins need to traverse to get to their subcellular destinations (Bentley and Banker, 2016). The polarized distribution of proteins in the dendritic and axonal compartments is essential to maintaining neuronal homeostasis, which is highly relevant for neuronal chemical and electrical communication throughout the CNS (Bentley and Banker, 2016). Furthermore, during CNS development, neuronal polarization is also essential, as neuronal migration and positioning within the developing brain ultimately impact neuronal connectivity (Bentley and Banker, 2016). One of the first relevant processes in neuronal polarization is evidenced by the change in localization and polarity of the GC, in a highly regulated process that is essential for axonal specification and dendritic growth. The Serine/Threonine Kinase 25 (STK25) is a crucial mediator of GC polarity; STK25 downregulation inhibits axonal specification in hippocampal neurons, and conversely, overexpression of STK25 leads to the development of multiple axons (Santana and Marzolo, 2017). Golgi morphology is also significantly disrupted in STK25- silenced neurons, consistent with loss of dendritic polarity of the GC (Matsuki et al., 2010). STK25 expression is also a determinant for establishing neuronal polarity and appropriate migration in developing mouse neocortex and hippocampus (Matsuki et al., 2013; Rao et al., 2018). Additionally, the knockdown of STK25-interacting partners, the pseudokinase STE20-Related Kinase Adaptor (STRAD), Liver kinase B1 (LKB1) and Golgi matrix protein 130 kDa (GM130) reproduces such polarity and migration deficits (Matsuki et al., 2010, 2013; Orlova et al., 2010), suggesting a pivotal role for the STK25-STRAD-GM130-LKB1 complex, in GC polarity and neuronal development.

As Golgi positioning determines neuronal polarity, dendritic, and axonal development show differential reliance on secretory trafficking (Ye et al., 2007). For instance, limiting ER-to-Golgi trafficking decreases membrane supply toward the dendrites without affecting the axon (Ye et al., 2007). More importantly, secretory membrane trafficking and dendritic development are highly dependent on discrete units of ER and GC located within the dendritic compartment which fulfill essential functions in protein synthesis, maturation and sorting to target membranes (Hanus and Ehlers, 2008).

Endoplasmic reticulum-related structures referred to as ER exit sites (ERES) and ER-Golgi intermediate compartments (ERGICs) are widely distributed throughout the soma and dendritic compartment, in stark contrast to discrete Golgi units referred to as Golgi outposts (GOPs) which are mostly found in proximal dendrites of cultured hippocampal neurons and in apical dendrites of pyramidal neurons *in vivo* (Ramírez and Couve, 2011). Several protein markers for *cis-*, *medial-*, and *trans*-Golgi have been identified in GOPs. However, their abundance and colocalization with one another are highly dynamic, suggesting that GOPs form by discrete Golgi compartments which only transiently interact with one another (Zhou et al., 2014), distinct from the classical GC ribbon morphology observed in non-polarized cells or neuronal soma.

Dendrites containing GOPs exhibit higher complexity compared to the ones lacking these structures. Furthermore, dendritic polarization of the somatic Golgi precedes the increase of dendritic complexity (Horton et al., 2005). The frequent location of GOPs at dendritic branch points, in conjunction with time-lapse analysis, showed that dendritic branches dynamically extend as GOPs move toward a developing branch (Ye et al., 2007). Finally, disruption of Golgi polarity by overexpression of Golgi reassembly-stacking protein of 65 kDa (GRASP65), a binding partner of GM130, leads to loss of dendritic arbor polarity and uniform growth of dendritic branches (Horton et al., 2005).

Golgi outposts are not only important for dendritic development; in fact, proteins associated with synaptic function also traffic through these distal GC structures. For instance, the kainate receptor GluK2 readily colocalizes to GOPs 30 min after synchronous release from the ER, and activation of protein kinase C (PKC) reduces GluK2 ER exit (Evans et al., 2017). In contrast, PKC-dependent phosphorylation of synaptic scaffold synapse-associated protein 97 (SAP97) protein is necessary for interacting with ADAM10 and facilitating its exit from GOPs into the synaptic compartment (Saraceno et al., 2014). Trafficking of *N*-methyl-D-aspartate (NMDA) receptors also bypasses somatic GC and localizes to GOPs where it colocalizes with SAP97 (Jeyifous et al., 2009). Finally, specialized GOPs enriched in glycosylation machinery and positive for the TGN resident protein calneuron-2 (Mikhaylova et al., 2009) referred to as Golgi satellites (GS), have been described to be widely distributed in the dendritic arbor and only occasionally associated with GC cisternae markers (Mikhaylova et al., 2016). Interestingly, the motility of GS is substantially reduced upon treatment with the GABA receptor antagonist bicuculine and with BDNF treatment, suggesting a regulatory role for these GS in activity-dependent neuronal plasticity (Mikhaylova et al., 2016). Indeed, GS colocalized with synaptic transmembrane proteins including synaptic cell adhesion proteins Neuroligin-1 and Neuroplastin-55, synaptic scaffold protein Homer1 and ionotropic receptor subunit GluA1 (Mikhaylova et al., 2016). The role of GOPs in synaptic plasticity has been controversial as they are mostly found in the largest dendrite of pyramidal neurons; however, the description of specialized and widely distributed GS which readily colocalize with synaptic components provides a fresh impulse to this idea and warrants further investigation.

Despite the pivotal role of GOPs in neuronal function and development, we know little about their biogenesis. Live-cell imaging experiments have shown that GOP biogenesis at apical dendrites requires GC deployment from the soma and subsequent tubule fission which involves the activation of GC-localized RhoA GTPase and downstream effectors, LIM motif-containing protein kinase 1 (LIMK1) and cofilin (Quassollo et al., 2015). These data suggest that GOPs generate from an existing GC cisterna rather than synthesized *de novo* at the dendritic compartment and depends on the regulation of the actin cytoskeleton. More recently GOPs biogenesis was determined to be highly dependent on the secretory pathway, as silencing of Sec23 and Sec31, components of the COPII complex mediating anterograde transport from the ER to the *cis*-Golgi (Fromme et al., 2008), decreases the number of GOPs in *Drosophila* Dendritic arbor (Da) neurons (Chung et al., 2017). Moreover, expression of these subunits is enhanced by CrebA expression (CREB3L in mammals), a master transcriptional regulator of secretory trafficking-associated genes (Fox et al., 2010). Accordingly, CrebA overexpression in *Drosophila* Da neurons increased GOP abundance in dendrites (Chung et al., 2017).

To sum up, the highly polarized nature of neurons is highly dependent on GC positioning. Furthermore, long-range protein synthesis and transport are mediated by discrete units of GC such as GOPs and GS. Over the next sections, we will discuss how the disruption of physiological GC function directly impacts in neurological disorders.

## Structural Proteins of the GC and Their Role in Neurodevelopment and Disease

Golgins are a set of proteins characterized by the presence of coiled-coil domains that play a substantial role in maintaining GC morphology (Muschalik and Munro, 2018). Golgins associate with several proteins including small GTPases of the Rab and Arl families, that control their tethering function and membrane recruitment (Cheung and Pfeffer, 2016). Loss-of-function approaches targeting several Golgins have proven their role in maintaining GC architecture in cell culture systems, but little is known of their role in the development and function of the nervous system or neuropathology.

Animal knockout (KO) models for Golgins such as Giantin or GMAP 210 have shown them to be essential for healthy development, as these animals exhibit defects in craniofacial and skeletal development (Smits et al., 2010; Stevenson et al., 2017). On the other hand, KO of Golgin-84, a protein closely related to Giantin, does not show any significant developmental abnormalities, and compound mutants for both of these Golgins do not show any additional defects (McGee et al., 2017). While these models highlight the importance of Golgins in embryonic development, some Golgins might be dispensable; indeed, several Golgins are associated with human diseases (Toh and Gleeson, 2016), but only a few have been linked to obvious neurological defects.

In this regard, neuronal GM130 KO mice showed severe motor defects similar to ataxia (Liu et al., 2017). GM130 is one of the most studied Golgins and is known to maintain Golgi ribbon morphology (Lowe et al., 1998). Furthermore, GM130 is necessary for maintaining the interaction of multi-cisternae structures within distal dendrites in *Drosophila* (Zhou et al., 2014). GM130 KO in mice leads to Purkinje cell degeneration in the cerebellum and impaired secretory trafficking. The latter ultimately affects dendritic growth, as previously described in *Drosophila* (Zhou et al., 2014; Liu et al., 2017), and these defects were both associated with Golgi fragmentation and abnormal positioning (Liu et al., 2017). On the other hand, GM130 overexpression is observed in *in vitro* models for mucopolysaccharidosis type IIIB (MPSIIIB) (Roy et al., 2011), a lysosomal storage disorder featuring strong neurological symptoms such as intellectual disability and progressive dementia (Kan et al., 2014). Most notably, overexpression of GM130 alone mimicked MPSIIIB cellular defects observed in HeLa cells (Roy et al., 2011). In conclusion, it appears both gain- and loss-of-function of GM130 drastically impact the morphology and function of the GC and could be a promising target for pharmacological intervention.

Mutations in the Golgin bicaudal D homolog 2 (BICD2) has been described in spinal muscular atrophy related disorders (Neveling et al., 2013). BICD2 participates in vesicular trafficking through direct interaction with dynactin and dynein motor proteins (Splinter et al., 2012) in association with active Rab6 (Cheung and Pfeffer, 2016). Overexpression of BIDC2 missense mutants affecting the coiled-coil domain, in HeLa cells, leads to severe GC fragmentation (Neveling et al., 2013). Most notably, the expression of these mutants in neurons leads to increased motility of BICD2/dynein–dynactin complexes and reduced neurite outgrowth (Huynh and Vale, 2017).

**FIGURE 1:**
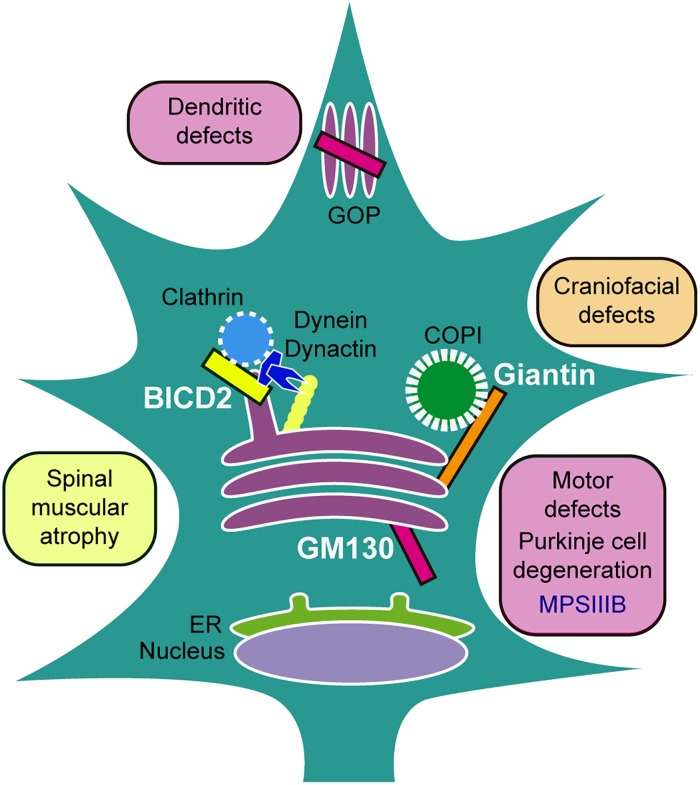
Golgins implicated in neurodevelopment and disease. BICD2 usually found at TGN, interacts with dynein/dynactin controlling vesicular trafficking through microtubules; mutations are related to Spinal muscular atrophy disorders. Giantin tethers COPI vesicles to the GC; the *knockout* animals manifest craniofacial defects. GM130 localizes in *cis-*Golgi and GOPs, participates in Golgi positioning and is involved in the maintenance of Golgi ribbon morphology and multi-cisternae structures; *knockout* animals display motor defects, cerebellar Purkinje cell degeneration, and aberrant dendritic growth, while overexpression is linked with a model of the lysosomal storage disorder Mucopolysaccharidosis type IIIB (MPSIIIB).

In conclusion, GC structural proteins play important roles in development and disease; however, there remains much to elucidate regarding the direct role of Golgins in inheritable neurological disorders and neurodevelopment (Figure 1).

## Disruption of GC Architecture in Neurological Disorders

### Alzheimer’s Disease

Alzheimer’s disease is a neurodegenerative disorder featuring progressive neuronal deterioration and cognitive impairment (Verheijen and Sleegers, 2018). It is widely accepted that AD arises from abnormal proteolytic processing of the amyloid precursor protein (APP), which leads to the generation of Aβ peptides, whose aggregation and deposition enhances neuronal cytotoxicity (Ittner and Götz, 2011). Aside from the amyloid cascade hypothesis, the appearance of neurofibrillary tangles of hyperphosphorylated Tau, a protein commonly associated with the microtubule network, is also a common histopathological observation in AD (Ittner and Götz, 2011).

Interestingly, disruption of GC architecture is observed in neuropathological processes associated with AD. There is growing interest in studying these phenomena in neurodegenerative disorders, as GC fragmentation is thought not to be the result of apoptosis or cell death associated pathways but rather precede them (Nakagomi et al., 2008).

**FIGURE 2:**
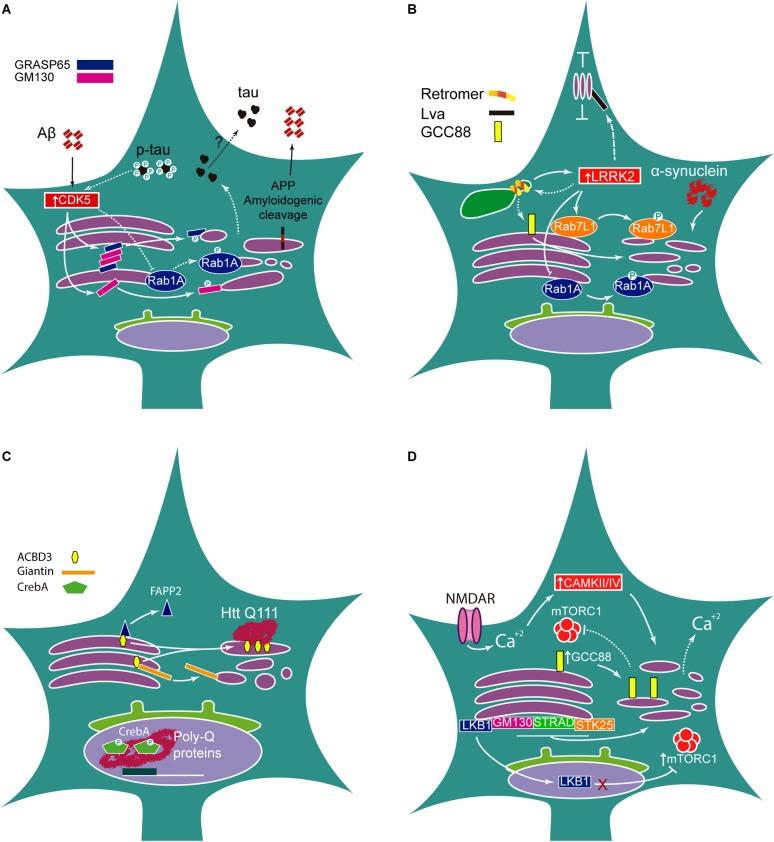
Golgi morphology in neurological disease. **(A)** In AD, accumulation of Aβ peptide activates CDK5 that phosphorylates and inhibits Golgi structural proteins GRASP65 and GM130 leading to Golgi fragmentation, which, in turn, promotes amyloidogenic proteolytic cleavage of APP. Pathogenic hyperphosphorylated Tau is linked to Golgi fragmentation via Rab1A inactivation by CDK5. On the other hand, Golgi disruption also increases Tau secretion, although the mechanism is unclear. **(B)** α-Synuclein overexpression leads to Golgi fragmentation. Enhanced LRRK2 kinase activity in PD mutations (LRRK2 G2019A) increases the phosphorylation of its targets Rab1A and Rab7L1, leading to Golgi disassembly. Mutations in the Retromer subunit VPS35 increase LRRK2 activity. In turn, LRRK2 G2019A could decrease VPS35 expression. *Knockdown* of another Retromer subunit, VPS26, induces Golgi disruption possibly involving GCC88. In *Drosophila*, the LRRK2 homolog, Lrrk, binds to Lva, decreasing motility of GOPs. **(C)** Mutant HTT interacts with overexpressed ACBD3, preventing ACBD3 binding to Giantin and leading to Golgi fragmentation. Mutant HTT also disrupts ACBD3 interaction with FAPP2 ending in mislocalization of FAPP2 to the cytoplasm. In *Drosophila*, Poly-Q proteins accumulate in the nucleus and inhibit CrebA, obstructing gene regulation. **(D)** The electrical activity, particularly increased in Epilepsy, induces Calcium-dependent CAMKII/IV activation, which leads to Golgi fragmentation; in turn, Golgi morphology could influence Ca^+2^ homeostasis. Loss of GM130, STRAD or STK25 results in Golgi disassembly. Mislocalization of LKB1 to the nucleus prevents phosphorylation of mTORC1, thus increasing mTORC1 activity. Nevertheless, slight overexpression of GCC88 induces Golgi fragmentation and could decrease mTORC1 activity.

The integrity of GC morphology regulates APP processing and has been extensively reviewed elsewhere (Joshi et al., 2015). Aβ triggers GC fragmentation through activation of cyclin-dependent kinase-5 (CDK5), which phosphorylates GRASP65. In turn, loss of Golgi integrity increases Aβ production by enhancing amyloidogenic protein cleavage (Joshi et al., 2014). Interestingly, activation of CDK5 by itself increases GC fragmentation through direct phosphorylation of GM130 (Sun et al., 2008). Moreover, overactivation of this kinase is common in AD and other neurodegenerative disorders including PD and ALS (Qu et al., 2007; Shukla et al., 2012; Klinman and Holzbaur, 2015). Overexpression of GRASP65 rescues GC morphology and increases accumulation of APP in the GC compartment, which, strikingly, is accompanied by an increase in non-amyloidogenic processing (Joshi et al., 2014). Indeed, more recently, the TGN has been identified as a major site for α-secretase cleavage and inhibition of APP trafficking from the TGN has been found to increase sAPPα levels (Andersen et al., 2005; Tan and Gleeson, 2019). Thus, GC integrity regulates proteolytic cleavage of APP in physiological and pathological settings (Figure 2A).

Golgi Fragmentation is induced in cell culture by overexpression of *wild type* (WT) and AD-associated mutant Tau (Liazoghli et al., 2005). Besides, the presence of neurofibrillary tangles in AD patients’ brains correlates with abnormal GC morphology and decreased surface area in the neocortex and hippocampus (Antón-Fernández et al., 2017). The increased neuronal activity triggered by KCl-mediated neuronal depolarization induces GC fragmentation by a CDK5-dependent mechanism (Mohamed et al., 2017). Concomitantly, KCl-induced depolarization increases secretion of Tau protein into the extracellular medium in WT neurons, which is inhibited by pharmacological blockade of CDK5, indicating that GC maintenance is a crucial regulator of Tau secretion (Mohamed et al., 2017). In this context, knockdown of the small GTPase Rab1A, which mediates ER to GC trafficking and whose deficiency is known to induce Golgi fragmentation (Haas et al., 2007), also increases Tau secretion independently of hyperexcitability (Mohamed et al., 2017). This correlation between neuronal hyperexcitability and Tau deposition is of particular interest since seizures are often comorbid with AD. Furthermore, hyperphosphorylated Tau can be detected in brains of patients with refractory epilepsy and AD patients with seizures show an accelerated cognitive decline (Tai et al., 2016; Vossel et al., 2016).

Altogether these data highlight the importance of Golgi morphology maintenance and its overall impact on the deposition of both Aβ peptide and Tau in the extracellular medium which, in turn, may enhance the spreading of cytotoxicity throughout the brain (Pignataro and Middei, 2017; Yamada, 2017). Thus, understanding the cellular basis of GC fragmentation could inform the development of drugs to slow down AD progression.

### Parkinson’s Disease

Parkinson’s Disease (PD) is the second most common neurodegenerative disorder after AD and is diagnosed based on motor abnormalities such as rigidity, bradykinesia, abnormal posture, and tremors (Błaszczyk, 2016). Determining the genetic basis for PD has been an elusive task. Nevertheless, a few genes have been linked to familial type PD including the encoding of the extensively studied α-synuclein (SCNA) and leucine-rich repeat kinase 2 (LRRK2) (Wood-Kaczmar et al., 2006).

Overexpression of α-synuclein in rat substantia nigra leads to GC fragmentation, a phenotype that is reversed by co-expression of Rab1A, which enhances ER to GC trafficking (Haas et al., 2007) (Figure 2B). While Rab1A expression is not able to rescue the neurodegeneration completely, it improves motor function in affected animals (Coune et al., 2011). Most notably, LRRK2 interacts and phosphorylates Rab1A, a phosphorylation event that is significantly induced by LRRK2 G2019A, a PD-associated mutant with increased kinase activity (Jeong et al., 2018). Additionally, overexpression of phospho-dead Rab1A induces neurodegeneration in cultured neurons (Jeong et al., 2018). However, despite these observations, the regulatory role of Rab1A phosphorylation remains unclear. LRRK2 also phosphorylates and directly interacts with Rab7L1, a TGN resident Rab GTPase that has also been identified as a candidate gene for PD (Fujimoto et al., 2018). LRRK2-mediated phosphorylation of Rab7L1 increases TGN fragmentation and PD-associated LRRK2 mutants demonstrate highly increased Rab7L1 phosphorylation rates (Fujimoto et al., 2018).

The retromer complex (VPS35-VPS26-VPS29), which participates in retrograde cargo retrieval from endosomes back to the TGN and recycling cargo back to the plasma membrane, has also been associated with PD (Zhang et al., 2018). Knockdown of VPS26 induces GC fragmentation (Seaman, 2004) and non-synonymous mutations in this subunit have been reported in PD patients (Gustavsson et al., 2015). This fragmentation is reduced by downregulation of the Golgin GCC88, which finely regulates GC morphology through interaction with the actin cytoskeleton (Makhoul et al., 2019). The retromer complex also appears to have a direct correlation with LRRK2 function, for instance, the brain tissue of patients featuring LRRK2 PD-associated mutations show reduced expression of retromer complex component VPS35 (Zhao et al., 2017). More interestingly, expression of PD-associated VPS35 D620N mutant elevates phosphorylation of Rab GTPases (Namely Rab8A, Rab10, and Rab12) and increases overall kinase activity of LRRK2 to a greater degree than PD pathogenic mutations (Mir et al., 2018).

Overall, these data point to an extensive regulatory network where proper functioning of LRRK2 kinase activity can modulate traffic between the GC and the ER through regulation of Rab GTPases. Furthermore, retrograde trafficking toward the GC in association with the Retromer complex is also affected by LRRK2 activity, thus regulating membrane protein availability and affecting the overall organellar organization.

Overexpression of LRRK2 G2019A is known to reduce dendritic arborization (Lin et al., 2010), which suggests that this protein might play a role in GOP biogenesis or function. Lrrk (*Drosophila* homolog of LRRK2) colocalizes with *medial* Golgi marker α-mannosidase II-GFP compartments in both the soma and dendrites of *Drosophila* Da neurons (Lin et al., 2015). Notably, Lrrk-positive GOPs are mostly stationary while other GOPs exhibit dynamic anterograde and retrograde movement within dendrites (Lin et al., 2015). The direct interaction of Lrrk with Lva, a golgin in *Drosophila* with no known orthologs (Sisson et al., 2000), prevents the association of GOPs to dynein–dynactin complexes, thus reducing motility. Consistently, decreased expression of Lrrk reduced GOPs anterograde movement and increased dendritic development (Lin et al., 2015). Remarkably, expression of LRRK2 G2019A is associated with an enhancement of retrograde GOPs movement, which is consistent with a suppressed dendritic arborization seen in neurons expressing this variant (Lin et al., 2015). In conclusion, LRRK2 function not only modulates GC structure but also plays an essential role in regulating GOPs positioning in maturing dendrites, which could underlie dendrite degeneration seen in animal models expressing mutant LRRK2.

The dysregulation of LRRK2 has been extensively studied and directly linked to mitochondrial dysfunction, oxidative stress, and synaptic dysfunction among others (Li et al., 2014). On the other hand, over this section, we have enlisted a wide array of protein interactors and phosphorylation targets which point out to an LRRK2 signaling hub residing at the GC that can directly impact on the organelle dynamics.

### Huntington’s Disease

Huntington’s disease (HD) is a neurodegenerative disorder characterized by progressive motor and cognitive impairment (Munoz-Sanjuan and Bates, 2011). It is genetically linked to an abnormal CAG repeat expansion in the first exon of *HTT*, a gene encoding the protein huntingtin (HTT), which participates in several cellular pathways including secretory and endosomal trafficking, transcriptional regulation, autophagy, and ciliogenesis (Saudou and Humbert, 2016). CAG repeats result in a poly-glutamine extension in the translated protein product, which is prone to aggregation and generates cellular toxicity (Labbadia and Morimoto, 2013). HD is one of several so-called poly-Q diseases, each one associated with a different gene locus but sharing similar pathological outcomes (Nath and Lieberman, 2017).

The Golgi adaptor acyl-coenzyme A binding domain protein 3 (ACBD3) is a GC resident protein which interacts directly with giantin and regulates GC morphology (Sohda et al., 2001). ACBD3 is overexpressed in the striatum of HD patients and in HD model mice where it interacts directly with mutant HTT (Figure 2C). The treatment of Q7 and Q111 striatal cell lines with monensin, an ionophore that disrupts acidic organelles including the GC and lysosomes used to model the Golgi stress response, increases ACBD3 levels in both cell lines with a greater induction in Q111, the cell line with higher polyglutamine repeats in HTT (Sbodio et al., 2013).

ACBD3 has also been implicated in glycosphingolipid synthesis in the GC through direct interaction with the transporter of glucosylceramide precursors, human four phosphate adaptor protein 2 (FAPP2) (Liao et al., 2018). Knockdown of ACBD3 leads to mislocalization of FAPP2 from the TGN to the cytoplasm, fragmenting the GC and severely affecting cellular lipidic profile (Liao et al., 2018). Glycosphingolipid metabolism has received considerable attention regarding neurological diseases such as HD, ALS, hereditary spastic paraplegia, lysosomal storage diseases, among others (Desplats et al., 2007; Robert et al., 2009; Boukhris et al., 2013; Dodge et al., 2015).

Metabolic stress related to amino acid deprivation is associated with HD. Low levels of cysteine γ-lyase (CSE), the enzyme directing cysteine biosynthesis has been documented in HD. Cysteine depletion triggers a stress response that enhances ATF4 expression, which is also reduced in HD and ultimately leads to increased oxidative stress and cytotoxicity (Sbodio et al., 2016). ATF4 is a stress-induced transcription factor that directs the expression of adaptative genes necessary to withstand stressors such as hypoxia, amino acid deprivation and organelle stress (Wortel et al., 2017). Q111 cells are highly sensitive to low cysteine medium and show reduced expression of both ATF4 and CSE. However, pre-treatment with low dose monensin rescues these expression levels and increases cell survival rates relative to control Q7 cells, without affecting GC morphology (Sbodio et al., 2018). Neurodegeneration in HD is a result of multiple stress pathways which can be successfully modulated with monensin and are also related to the expression of GC structural proteins, thus suggesting an important role for GC integrity in HD.

More recently, expression of poly-Q-containing Machado-Joseph’s protein associated with spinocerebellar ataxia type 3 (Matos et al., 2018) in Da neurons led to reduced dendritic branching and severe reduction of Mannosidase II-EGP positive GOPs in dendrites (Chung et al., 2017). This effect correlates with diminished expression of genes linked to the secretory pathway, including a master transcriptional regulator of secretory trafficking genes, CrebA (Fox et al., 2010; Chung et al., 2017). Indeed, upregulation of CrebA rescues GOP deficiency in poly-Q expressing Da Neurons (Chung et al., 2017). Nuclear localization of poly-Q containing proteins is known to disrupt normal transcriptional programming and perturb nuclear protein turnover (Mohan et al., 2014). However, its potential role in regulating secretory trafficking in neurons through transcriptional repression opens a new door for understanding the cellular pathways involved in poly-Q diseases.

As described above, HD and poly-Q diseases feature a wide array of pathological pathways ranging from metabolic regulation toward basic transcriptional activity issues. Nevertheless, the convergence of these pathways toward GC dynamics regulation offers a novel approach for understanding and ultimately treat these neurodegenerative disorders.

### Epilepsy

Epilepsy is a condition associated with recurrent, unprovoked seizures which result from a hypersynchronous discharge of neurons in the brain (Stafstrom and Carmant, 2015). The delicate balance between inhibitory and excitatory transmission is often disrupted, and most of the genes associated with epilepsy are known to regulate neuronal connectivity, synaptic discharge, and signaling, as well as ion channel function and trafficking (Meisler and Kearney, 2005; Poduri et al., 2013; Maljevic et al., 2019).

Neuronal hyperexcitability has been shown to regulate the GC, as chronic exposure to slightly elevated concentrations of KCl (5 nM) inducing neuronal depolarization for 2 days leads to increased GC fragmentation (Thayer et al., 2013). Additionally, pharmacological hyperactivation of neuronal activity through treatment with bicuculine or APV, GABAA, and NMDA receptor antagonists induces Golgi fragmentation (Thayer et al., 2013) (Figure 2D). Remarkably these changes in GC architecture could be reversed after returning neurons to the normal culture medium (Thayer et al., 2013), showing that GC structure is highly dynamic and remodels itself upon external pharmacological signals. Bicuculine-induced Golgi fragmentation can be prevented by pre-treatment with Ca^2+^/calmodulin-dependent protein kinase II/IV (CAMKII/IV) inhibitors, suggesting a high dependence of calcium signaling pathways in activity-induced GC fragmentation (Thayer et al., 2013). Several voltage-gated calcium channels associate with epilepsy (Rajakulendran and Hanna, 2016) and calcium signaling mediated by CAMKII has been consistently associated with neurological disease including epilepsy, schizophrenia, and autism spectrum disorders (Robison, 2014). Calcium homeostasis also relies on GC integrity in concert with ER and mitochondrial reservoirs (Lissandron et al., 2010; Yang et al., 2015). However, little is known of the role of GC calcium storage in cellular homeostasis or disease.

Golgi positioning defects could also play a role in the onset of seizure disorders, as the proliferation, migration, and differentiation of neural progenitors are ultimately responsible for shaping neuronal connectivity in the developing brain. Mutations in STRAD, a core protein in the Golgi positioning complex, were identified through microarray analysis in patients with polyhydramnios megalencephaly and symptomatic epilepsy (PMSE) syndrome (Puffenberger et al., 2007). Expression of a PMSE-associated 180-amino acid, C-terminal truncating mutation of STRAD in adult neuronal progenitor cells leads to the disassembly of the GC, reduced expression of GM130 and severe defects in dendritic development (Rao et al., 2018). The reported defects in dendritic growth were similar to the ones observed in STK25-deficient neurons. Furthermore, the knockdown of GM130 in adult neuronal progenitors reproduced this observation (Rao et al., 2018), suggesting an underlying role of GC integrity in the onset of this rare neurodevelopmental disorder.

The mammalian target of rapamycin (mTOR)-related signaling pathway has acquired increasing importance in seizure disorders, as somatic mutations of upstream and downstream signaling components, underlie cortical lamination deficits and the onset of focal cortical dysplasia and hemimegalencephaly, both neurodevelopmental disorders associated with seizures (Poduri et al., 2013). Most notably, a recent study pointed out that GC fragmentation associated with slight overexpression of Golgin GCC88 decreases mTOR activity and increases autophagy in HeLa cells (Gosavi et al., 2018), suggesting that disruption of GC morphology could lead to reduced mTOR activity in neurological disorders. Conversely, human PMSE brains and brains from STRAD knockdown mice show an enhanced activity of mTORC1 and cortical heterotopias resembling mTORC1-deficient lamination defects (Orlova et al., 2010). Increased mTORC1 activity was associated with LKB1 mislocalization to the nucleus (Orlova et al., 2010), a protein kinase which inhibits mTOR activity through activation of AMPK (Shaw et al., 2004).

Epileptic encephalopathies are highly heterogeneous disorders, and there is little understanding of the impact of genetically associated variants on phenotypical severity (Niestroj et al., 2018). Moreover, the availability of several pharmacological and genetic approaches both *in vivo* and *in vitro* can often lead to conflicting interpretations; however, we can conclude that both approaches ultimately lead to GC fragmentation associated to increased synaptic activity. Additionally, the direct role of the GC in regulating mTOR activation, a protein kinase consistently associated with epilepsy, gives us new insight for treating so-called “mTORopathies” (Crino, 2015). In this context, mTOR inhibitor, rapamycin has been successfully used to attenuate seizures and cortical dysplasia in animal models featuring enhanced mTOR activity (Hsieh et al., 2016).

## Reelin Signaling Regulates GC Dynamics and Morphology

In the next section, we will analyze the role of Reelin signaling in the regulation of these processes commonly affected in neurological disorders, and how this pathway plays an important role in the structuring and dynamics of the CG. Indeed, Reelin signaling dysfunction has been documented in several neurodevelopmental and neurodegenerative disorders including AD, PD, HD, and epilepsy (Haas et al., 2002; McCullough et al., 2012; Bodea et al., 2014; Baek et al., 2015; Cuchillo-Ibanez et al., 2016). Furthermore, Reelin signaling plays a central role in regulating neuronal migration and polarity during development and also regulates neuronal plasticity in the adult brain (Santana and Marzolo, 2017; Wasser and Herz, 2017).

In this context, Reelin was first described as a protein absent in the mouse mutant *reeler* which features defective neuronal migration in the neocortex and hippocampus, resulting in the inversion of cortical layering (Frotscher, 2010). Most notably, *reeler* recapitulates phenotypes observed in GM130 KO mice (Liu et al., 2017) including ataxia and cerebellar atrophy (Yuasa et al., 1993; Magdaleno et al., 2002) suggesting that Reelin participates in pathways associated with Golgi structural regulation during early brain development.

**FIGURE 3:**
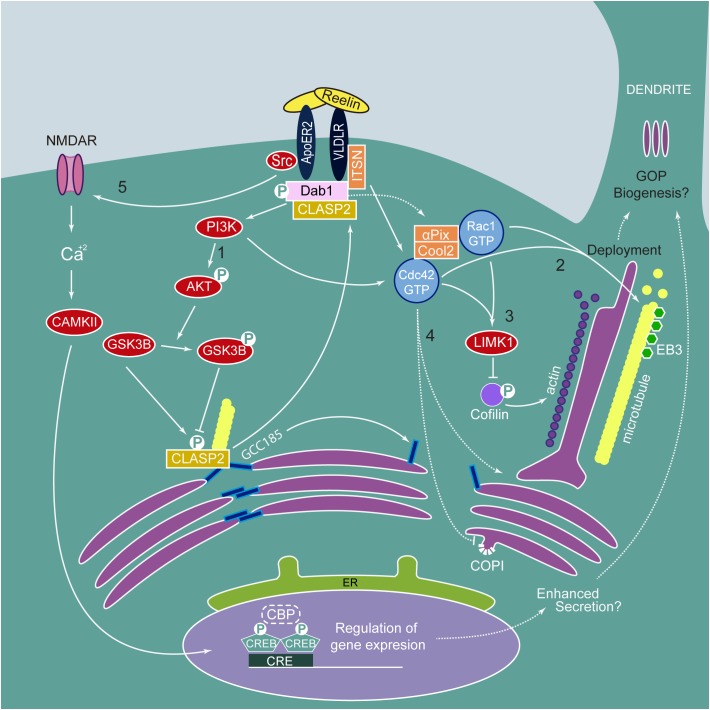
Reelin signaling pathway in Golgi Dynamics and morphology. Reelin binds its receptors ApoER2 and VLDLR triggering the phosphorylation of Dab1 by Src Family Kinases. pDab1 activates PI3K/AKT resulting in (1) phosphorylation and inactivation of GSK3β. Inactivated pGSK3β is not able to phosphorylate CLASP2, releasing it from GCC185. Unphosphorylated CLASP2 can bind pDab1 and GCC185, not bound to the microtubules, leads to dismantling of Golgi ribbon morphology. pDab1 via PI3K and ITSN activates Cdc42. The Cdc42/Rac1 GEF αPix/Cool2 has been involved in Reelin-induced Golgi translocation after axon specification (not shown in the figure) and deployment to the apical dendrite. (2) Active Cdc42 and Rac1 induce the positioning of microtubule-associated Golgi vesicles possibly via regulation that involves dynein and microtubule-associated Lis1, necessary for Golgi translocation and deployment. Besides, Reelin signaling promotes the formation of dynamic plus end microtubules labeled with EB3. (3) Also, Cdc42/Rac1 activate LIMK1 that phosphorylates and inactivates cofilin, leading to actin fiber formation, required for Golgi deployment and possibly GOPs formation. (4) Furthermore, active Cdc42 could regulate Golgi transport by reducing COPI retrograde trafficking and promoting tubular Golgi formation at the TGN. (5) Src also phosphorylates NMDAR, increasing Ca^2+^ influx and activating CAMKII, which phosphorylates CREB and, together with CBP regulates gene expression, thus, conceivably inducing genes to promote enhanced secretion and possibly GOP biogenesis.

Reelin is a large, secreted glycoprotein that binds to the ApoE receptor 2 (ApoER2) and/or very low density lipoprotein receptor (VLDLR) leading to Src-family kinases Fyn- and Src-dependent tyrosine phosphorylation of the adaptor protein Dab1, which in turn regulates several downstream pathways, including PI3K-Rac1/Cdc42, PI3K-AKT-GSK3β, and Crk/CrkL-Rap1 among others (Figure 3) (Santana and Marzolo, 2017).

Through the PI3K-Cdc42/Rac1 pathway, Reelin is an upstream activator of LIMK1 and cofilin, which, as previously discussed, participate in GC tubule fragmentation (Quassollo et al., 2015). However, it is not yet known if Reelin induces the formation of GOPs. What has been established is that Reelin signaling plays a role in GC deployment and positioning (Figure 3). The GC appears condensed near the nucleus in Dab1 and Reelin KO neocortical pyramidal neurons as opposed to extended toward the apical layer in control neurons (Matsuki et al., 2010). Strikingly, acute treatment with Reelin-conditioned medium in cultured hippocampal neurons leads to rapid deployment of the GC into the largest neurite. Furthermore, GC deployment is significantly reduced by expression of STK25, suggesting opposing roles for Reelin- and STK25-mediated GC modification (Matsuki et al., 2010). In nocodazole-treated hippocampal neurons, Reelin specifically promotes the reconstitution of disrupted dendritic GC, but not somatic GC. Reelin also induces the formation of plus-end dynamic microtubules labeled with the microtubule plus end binding protein 3 (EB3) (Meseke et al., 2013a). Other studies have shown that the GTPases Rac1 and Cdc42 (via its GEF aPIX) are involved in GC translocation and deployment (Jossin and Cooper, 2011; Meseke et al., 2013b). Cdc42 is found in the plasma membrane and the GC where it is known to participate in determining cell polarity and regulation of the actin cytoskeleton. Activation of Cdc42 also leads to reducing retrograde transport from the GC and increased intra-GC anterograde trafficking via COPI vesicles (Farhan and Hsu, 2016). It remains unclear whether Reelin activation of Cdc42 impacts the processes of cisternal maturation, and retrograde or anterograde trafficking.

More recently, Reelin signaling modulation of the microtubule nucleating protein CLASP2 has further strengthened the role of this pathway in the regulation of neuronal cell polarity and migration through regulation of GC morphology (Dillon et al., 2017). CLASP proteins are found in the GC and act as acentrosomal nucleation sites for the microtubule network (Efimov et al., 2007). CLASP2 associates with the TGN resident Golgin GCC185, a protein known to be necessary for maintaining ribbon formation of the Golgi stack (Brown et al., 2011). The overexpression of CLASP2 enhances dendritic branching and Golgi condensation (Beffert et al., 2012), thus supporting an important role for CLASP2 in GC dynamics. In line with this evidence, CLASP2 function has been consistently associated with dendritic and axonal development, synaptic function, neuronal progenitor proliferation, and migration (Beffert et al., 2012; Dillon et al., 2017).

CLASP2 directly interacts with Dab1 and triggers phosphorylation by GSK3β (Dillon et al., 2017). CLASP2 features multiple GSK3β phosphorylation sites, allowing for a delicate tuning of microtubule binding activity which differentially affects dendritic and axonal growth (Hur et al., 2011). Furthermore, Reelin signaling increases inhibitory phosphorylation of GSK3β mediated by AKT (Beffert et al., 2002), adding to a complex regulation of microtubules through regulation of this kinase. GSK3β can be found in the TGN of HeLa cells, and its depletion causes Golgi fragmentation and deregulation of anterograde trafficking, diverting the cation-independent mannose-6-phosphate receptor, CI-M6PR, from transport to the pre-lysosomal compartment into the exocytic pathway (Adachi et al., 2010). GSK3β localization at the GC is dependent on TGN Golgin p230 and also determines CLASP2 phosphorylation status and localization, as GSK3β depletion leads to increased peripheral localization of CLASP2 (Adachi et al., 2010).

An exciting and recently described Reelin signaling modulator is the multi-scaffold protein Intersectin (ITSN), which acts as a guanine nucleotide exchange factor for Cdc42 and also functions as an essential scaffold for endocytic and exocytic processes at the pre-synapse, making it an interesting protein to study in pathological conditions associated with neurotransmitter release such as epilepsies (Gerth et al., 2017). Pharmacological inhibition of the interaction between ITSN and Cdc42 reduces GC motility and disrupts GC organization through modification of the actin cytoskeleton (Friesland et al., 2013). ITSN1 also interacts directly with Golgin GCC88 in HeLa cells and localizes to the TGN. Most notably, GCC88-induced GC fragmentation is significantly reduced in ITSN1-silenced HeLa cells, further suggesting a role for this protein in actin regulation (Makhoul et al., 2019).

ITSN1 and ITSN2 interact directly with VLDLR and Dab1, and Reelin-mediated Dab1 phosphorylation is significantly reduced in ITSN1 KO neurons abrogating Reelin-mediated long term potentiation (LTP) enhancement (Jakob et al., 2017) thus directly affecting learning and memory processes (Nicoll, 2017). Moreover, double KO of ApoER2 and ITSN1 show laminar defects reminiscent of ApoER2/VLDLR double KO (Jakob et al., 2017). While these findings directly implicate ITSN in Reelin signal transduction associated with neuronal migration and plasticity, the recent description of ITSN residing at the TGN opens the possibility for novel functions of this protein in Reelin-associated GC regulation. In conclusion, Reelin signaling regulates both the actin and microtubule cytoskeleton and in turn these accessory proteins directly impact on Reelin pathway activation.

Throughout this article, we have also argued for an essential role of neuronal activity-dependent modulation of GC architecture. Hyperexcitability is a major driver of GC fragmentation and may account for common morphological GC features observed in neuropathological conditions (Thayer et al., 2013). It is also interesting that increased synaptic activity modulates Reelin signaling. As already mentioned, it is well-established that Reelin signaling enhances LTP in hippocampal slices through binding of ApoER2 and increased recruitment of AMPAR to the cell surface (Weeber et al., 2002; Beffert et al., 2005; Qiu et al., 2006) and downstream activation of cAMP response element-binding protein (CREB) transcriptional activator (Telese et al., 2015). More recently, stimulation of synaptic activity by a short incubation with 10 mM KCl was shown to induce dendritic clustering of ApoER2 in hippocampal neurons without modifying its total expression (Pfennig et al., 2017). Increased activity also enhances Dab1 phosphorylation and localization at synaptic sites (Pfennig et al., 2017) suggesting that alterations in synaptic activity modulate Reelin signaling activation through ApoER2.

On the other hand, the secretion of Reelin itself appears independent of calcium-mediated exocytosis that is modulated by synaptic activity, although it is sensitive to brefeldin A, suggesting that this process requires a fully functioning GC (Lacor et al., 2000). Interestingly, the incubation of neurons with kainic acid, as an *in vitro* model for epileptic seizures, leads to GC dispersion and decreased proteolytic processing of Reelin evidenced by an increase in the full length 400 kDa protein and a decrease in the 320 kDa secreted Reelin. Furthermore, the glycosylation level of the 320 and 400 kDa secreted Reelin is significantly decreased in kainic acid-treated neurons (Kaneko et al., 2016). Altogether, these data suggest, that Reelin post-translational modifications and secretion might be sensitive to glutamate and kainate receptor agonists, and further suggests a regulatory role for neuronal excitability in Reelin pathway signal transduction.

Overall, Reelin signaling appears to impact GC dynamics during development directly and might play a role in the interplay between cell signaling and activity-dependent plasticity which also regulates the GC. Nevertheless, how Reelin signaling directly impacts secretory trafficking remains to be determined. In this regard, a few clues can be found through the examination of transcriptional regulatory pathways. Several lines of evidence point out that efficient secretory pathway functioning is essential for the establishment of GOPs in dendrites. Indeed, overexpression of CrebA in Da neurons increased the number of GOPs by upregulating genes associated with ER-Golgi anterograde trafficking (Chung et al., 2017). Most notably, CREB-binding protein (CBP) has been shown to interact directly with CrebA in *Drosophila* (Anderson et al., 2005) and CBP modulation also regulates GOP abundance (Chung et al., 2017).

CREB-binding protein and related transcriptional partners including CREB and p300 have been associated with Rubinstein–Taybi syndrome, a neurodevelopmental disorder within the autism spectrum (Roelfsema et al., 2005). CBP-dependent transcription is highly dependent on calcium influx, induces transcription of early genes associated with synaptic plasticity and is important for LTP in the hippocampus (Wood et al., 2005). Nevertheless, CBP also controls transcription in other cell signaling pathways. For instance, the recent identification of LRP8-Reelin-regulated neuronal enhancers for genes activated by acute treatment with Reelin showed that these are modulated by a transcriptional complex, which including CREB and CBP (Telese et al., 2015). Similarly, Reelin treatment enhances the transcription of early genes associated with neuronal plasticity in cortical neurons (Telese et al., 2015). While gene ontology analysis of Reelin-induced genes did not show enrichment in genes associated with protein trafficking, likely due to the acute nature of the stimuli, the convergence of related transcriptional activators further strengthens a possible role for Reelin signaling in the modulation of Golgi dynamics. Particularly interesting genes enhanced by Reelin treatment include the stress response effectors ATF4, PERK, and ETS2 (Telese et al., 2015). These genes are associated with transcriptional regulation induced by Golgi stress (Baumann et al., 2018; Sbodio et al., 2018), suggesting that Reelin-mediated transcriptional activation might precede major changes in Golgi protein composition and directly impact the secretory pathway.

Altogether we can conclude that Reelin signaling impacts GC dynamics through 4 distinct mechanisms: (1) The activation of LIMK1, Cdc42, and GSK3β all well-known downstream effectors of Reelin signaling. (2) The direct interaction and regulation of cytoskeletal scaffolds with core receptor components ApoER2, VLDLR, Dab1 which could suggest a structural role for these proteins at the GC, distinct from their role as Reelin receptors at the plasma membrane. (3) The regulation of synaptic plasticity which modulated GC dynamics in both physiological and pathological conditions (4) and finally, while there is a robust description of several protein kinases that become activated upon induction of the Reelin pathway, we have only scratched the surface of the transcriptional regulatory network activating downstream. In this context, future transcriptomic and proteomic approaches could unveil new components associating Reelin signaling with GC dynamics.

## Concluding Remarks

One hundred and twenty years have passed since the Golgi complex was first described, and a formidable body of information regarding its structure, protein composition, homeostatic dynamics, and trafficking has since been elucidated. Different from other cellular pathways, our basic knowledge of the dynamic regulation of the GC and its function in protein trafficking has preceded our knowledge of how defects in this organelle’s homeostasis affect health and disease. In this article, we have discussed the current information linking GC dysfunction with neurodegenerative and neurodevelopmental disorders. It is clear that GC dynamics is directly linked to common neuronal defects observed in neurological disorders including abnormal proteolytic processing, dendritic arborization, neuronal migration, and synaptic plasticity. While each of these processes encompasses a broader and more complex protein network, our review should prompt a more thorough investigation of the GC in the onset, diagnosis, and treatment of neurological disorders.

Expanding our knowledge in this area will significantly aid clinical investigation as genome-wide association studies and large-scale exome sequencing initiatives have consistently unveiled variants and protein networks directly associated with both endosomal and secretory trafficking pathways which ultimately converge in the GC (Nuytemans et al., 2013; Novarino et al., 2014; Raghavan et al., 2018; Striano and Nobile, 2018). More importantly, a large body of data directly points to a central role for cell signaling cues in the regulation of Golgi dynamics in both development and disease. Indeed, Reelin signaling has been implicated in neuronal polarity, migration and synaptic function, and its direct interaction with downstream effectors linked with the regulation of GC architecture places this pathway as a potential master regulator of GC biology and a pharmacological target. Indeed, chemical modulators for Cdc42, LIMK1, and GSK3β are currently available (Hong et al., 2013; Petrilli et al., 2014) and several FDA-approved GSK3β inhibitors are already being used in the treatment of neurological diseases (Caracci et al., 2016).

Finally, we have extensively discussed changes in GC architecture. However, there is little and often conflicting information about how Golgi disassembly or fragmentation affects integral trafficking of transmembrane proteins and ionotropic channels towards the cell surface or synaptic compartment. Protein translation and ER stress are well-known to be associated with neurological diseases. Here, we have comprehensively described the GC alterations in neuropathology. Future work connecting the role of the GC in neuropathology with its established participation in delivering protein cargo to organelles, endosomes and the plasma membrane, will consolidate our understanding of the secretory pathway and unveil novel cellular paradigms for understanding prevalent neurological diseases.

## Author Contributions

MOC wrote and designed the first draft of the manuscript and made a relevant contribution to the general idea of the review article. LMF made the figures and wrote the legend section of the manuscript. M-PM supervised all steps of the writing process, including the structure of the article and figures, the list of references, and wrote and edited the final version of the manuscript. All authors contributed to manuscript revision read and approved the submitted version.

## Conflict of Interest Statement

The authors declare that the research was conducted in the absence of any commercial or financial relationships that could be construed as a potential conflict of interest.
